# USING COMPANION CARE TO ADDRESS THE DUAL BURDEN OF LONELINESS AND CHRONIC ILLNESS

**DOI:** 10.1093/geroni/igae098.3764

**Published:** 2024-12-31

**Authors:** Kelsey McNamara, Jill Bongiorni Meadows, Brian Smith

**Affiliations:** Papa Inc., Brooklyn, New York, United States; Papa Inc., Miami, Florida, United States; Papa Inc., Miami, Florida, United States

## Abstract

Chronic illness is a risk factor for loneliness. Concurrently, loneliness can increase the risk of and exacerbate chronic conditions, especially among older adults. Special Supplemental Benefits for the Chronically Ill (SSBCI) are benefits that can be offered to qualifying Medicare Advantage members to improve health and function. This study’s objective was to evaluate the impact of a companion care program, designed to address loneliness and lack of social support, on SSBCI recipients’ quality of life. Members received companion care through their health plan and were paired with “Papa Pals” for companionship and assistance with instrumental activities of daily living. Members were assessed at enrollment and ≥8 weeks later for loneliness (UCLA Three-Item Loneliness Scale, Range: 3-9, =3 “not lonely”, 4-6 “lonely”, 7-9 “severely lonely”) and health-related quality of life (CDC’s Healthy Days Measure, Range: 0-30 days). Change in outcomes were analyzed using the one-sample t-test. Analysis included 531 SSBCI recipients who were lonely at baseline, used companion care any time between 2021-2023, and completed a reassessment. Average age was 74, 63% were female. Average baseline values: 5.7 loneliness score, 9.6 mentally unhealthy days, 14.1 physically unhealthy days. On average, outcomes improved: -0.99 loneliness score, -1.4 mentally unhealthy days, -1.6 physically unhealthy days. In regards to loneliness score, 66% of members improved, 13% maintained, 22% regressed. Our results indicate companion care can significantly improve loneliness among a population experiencing a high burden of physical and emotional stress; research should continue to evaluate scalable interventions to address loneliness among this population.

